# Peripheral immune signatures associated with the risk of hepatocarcinogenesis in cirrhotic Egyptian HCV patients before and after treatment with direct-acting antivirals

**DOI:** 10.1186/s12985-024-02551-3

**Published:** 2024-11-15

**Authors:** Reem El-Shenawy, Rehab I. Moustafa, Naiera M. Helmy, Yasmine S. El-Abd, Ashraf A. Tabll, Yasser K. Elesnawy, Heba Shawky

**Affiliations:** 1 Microbial Biotechnology Department, Biotechnology Research Institute, National Research Centre, Dokki, Cairo, 12622 Egypt; 2 School of Pharmacy, Newgiza University (NGU), Newgiza, 12577 Giza Egypt; 3 Egypt Center for Research and Regenerative Medicine (ECRRM), Cairo, Egypt; 4 National Committee for Control of Viral Hepatitis (NCCVH), Ministry of Health and Population, Cairo, Egypt; 5 Therapeutic Chemistry Department, Pharmaceutical Industries and Drug Research Institute, National Research Centre, Dokki, Cairo, 12622 Egypt

**Keywords:** HCV-related HCC, DAA, Claudin, sCD163, Cytokines, Tight junction proteins

## Abstract

**Background:**

Although direct-acting antivirals (DAAs) have revolutionized the management of chronic HCV, the debatable association with hepatocellular carcinoma (HCC) occurrence/recurrence has raised major concerns about their long-term use, especially in cirrhotic cases. The role of epithelial tight junction proteins (TJPs) in hepatocarcinogenesis has been highlighted; however, the association of their expression in peripheral blood mononuclear cells (PBMCs) with HCC has rarely been reported. This study aimed to explore the role of peripheral claudin *(Cldn)*1 in liver pathogenesis and its crosstalk with soluble immune mediators in HCC prognosis.

**Methods:**

The study population included six independent subgroups: healthy controls, cirrhotic/non-cirrhotic treatment-naïve HCV patients, DAA-SVR patients, and anticancer treatment-naïve *de novo* HCC patients. The laboratory tests included serum levels of alpha-fetoprotein (AFP), albumin, liver transaminases, total bilirubin, and CBC profiling. The serum levels of soluble cluster of differentiation (sCD)163, IL-10, and IL-12 were estimated by corresponding ELISA kits, whereas the levels of *Cldn1* and transforming growth factor (*TGF*)-*β* in PBMCs were quantified using quantitative PCR (qPCR).

**Results:**

Serum sCD163, IL-10, and IL-12 levels were significantly higher in the HCC patient group than in the control and non-malignant patient groups (*P* < 0.0001). No significant difference was detected in the serum levels of the three markers between cirrhotic and non-cirrhotic patients of chronic HCV, whereas their levels were significantly different between cirrhotic and non-cirrhotic SVRs (*P* < 0.0001). Similarly, the transcriptional levels of peripheral *Cldn1* and *TGF-β* were significantly higher in patients with HCC and non-malignant cirrhosis than in patients without cirrhosis (*P =* 0.0185–<0.0001 and 0.0089–<0.0001, respectively). Logistic regression analysis revealed a significant association between all the abovementioned markers and HCC (*P* = 0.0303 to < 0.0001), which was further confirmed by the results of receiver operating characteristic (ROC) analysis, which revealed an area under the curve (AUC) value ranging from 0.883 to 0.996. The calculated cutoff values demonstrated remarkable prognostic capacity, with ranges of 88–99.41% and 82.14–97.92% and positive/negative predictive values ranging from 84.62 to 98.3% and 92–98%, respectively.

**Conclusion:**

Serum sCD163, IL-10, IL-12 and peripheral *Cldn1* and *TGF-β* expression levels represent novel non-invasive HCC biomarkers that maintain their predictive power under different pathological conditions and circumvent the drawbacks of conventional prognostic markers in patients with mild cirrhosis and/or normal AFP, albumin, and/or platelet counts.

**Supplementary Information:**

The online version contains supplementary material available at 10.1186/s12985-024-02551-3.

## Introduction

The burden of hepatocellular carcinoma (HCC) is increasing worldwide as the third leading cause of cancer-related deaths, accounting for 90% of primary liver cancers and 8.3% of all cancer-associated mortalities, particularly among elderly people [1]. Several risk factors have been associated with HCC, including long-term exposure to fungal aflatoxins, smoking, alcohol abuse, metabolic diseases such as diabetes mellitus, non-alcoholic fatty liver disease (NAFLD), and chronic viral infections such as hepatitis B (HBV) and C (HCV) viruses [2]. In particular, HCV remains the most common cause, and in addition to underlying cirrhosis as a key precursor of HCC, viral antigens can induce several protumorigenic mechanisms, including persistent oxido-inflammatory-mediated liver damage and dysregulation of cellular signaling pathways [3]. Evidently, chronic HCV infection can be efficiently treated with direct-acting antivirals (DAAs) that achieve a sustained virologic response (SVR) in > 95% of patients, improving extra-hepatic manifestations and “presumably” reducing the risk of cirrhosis-associated HCC [4]. However, several clinical studies and real-life experiences have revealed that the subsequent complications and risk of HCC are not entirely eliminated in all recovered patients, especially in cirrhotic patients [5–9]. One reason for this could be related to the persistence of HCV biological imprints after SVR, including virus-induced epigenetic changes in the liver associated with HCC development [10]. Another possible explanation is that cirrhosis-induced alterations in the immune milieu are exacerbated by the introduction of DAAs and are similarly associated with a greater risk of HCC [11].

Considering the recent debate about the correlation between DAA-based therapy and HCC occurrence/recurrence [12] and the launching of nationwide surveillance programms for HCC in Egypt resembling those previously adopted for the testing and treatment of HCV [13], long-term follow-up for cirrhotic patients receiving DAAs is highly recommended. Typically, HCC prognosis and/or diagnosis often depend on several clinicopathological criteria, including tumor number, size, and focality; lymphovascular invasion, and pathological grade and stage [14]. In addition, the serum alpha-fetoprotein (AFP) level has long been a less invasive gold standard predictor of HCC [15]; however, the reliability of AFP may be compromised in patients with normal serum AFP levels, including HCC patients [16] and non-malignant cirrhotic patients [17], which necessitates the combination of other serum markers and imaging modalities [18].

Because HCC is an inflammation-induced cancer [19], various soluble immune mediators have been proposed as potential biomarkers for HCC prognosis, given their regulatory role in the host antitumor immune response, which determines the overall fate of tumorigenesis [20]. The tumor microenvironment (TME) is dominated by immunosuppressive cytokines that curtail the effector antitumor immune response and promote the survival and proliferation of tumor cells. Among these factors, transforming growth factor (TGF)-β and IL-10 are key players in HCC pathogenesis, where dysregulated TGF-β signaling pathways promote persistent liver inflammation, fibrogenesis, and TME immunomodulation [21]. Moreover, intrahepatic expression of the M2 cytokine IL-10 is the main modulator of liver fibrinogenesis during chronic infection [22] and one of the main precursors of HCC through genetic polymorphisms [23], which also contributes to perpetuating immunosuppressive conditions in the TME [20, 21]. Similarly, soluble CD163 (sCD163), a specific biomarker for M2 macrophage activation, has been reported to play a key role in liver pathogenesis by activating several immunoregulatory pathways and therefore was introduced as a useful tool for assessing the severity of liver damage and predicting HCC [24]. Although chronic inflammation induced by several proinflammatory mediators has recently been identified as a co-factor in carcinogenesis [25], the pleiotropic cytokine IL-12 plays an essential role in inflammation-associated tumorigenesis, despite its classical role in activating cytotoxic T lymphocytes (CTLs) and natural killer (NK) cells for tumor clearance [26].

In addition, tight junction proteins (TJPs) have been shown to play multiple roles in liver pathogenesis, ranging from viral entry to cancer progression and metastasis, by promoting epithelial–mesenchymal transition [27] and have therefore become important therapeutic targets for liver cirrhosis and carcinoma [28]. In a recent study, **Roehlen et al.** [29] proposed that epithelial *Cldn1* expressed on hepatocytes acts as a therapeutic target through specific antibodies that suppress protumorigenic cellular signaling and reprogram the TME [29]. Considering that blood samples are less invasive and easier to retrieve than tumor tissues are, peripheral blood mononuclear cells (PBMCs) could be an excellent alternative for tumor surveillance, especially because their transcriptome is of single origin and can efficiently reflect local immune cell infiltration in the TME [30]. Nonetheless, to our knowledge, only one machine learning-based study has recently investigated peripheral TJP transcription in liver cancer [31]. In this study, we aimed to explore the role of peripheral *Cldn1* expressed in PBMCs in liver pathogenesis and its crosstalk with soluble immune mediators in HCC prognosis.

## Experimental

### Study population

**Fig. 1:**
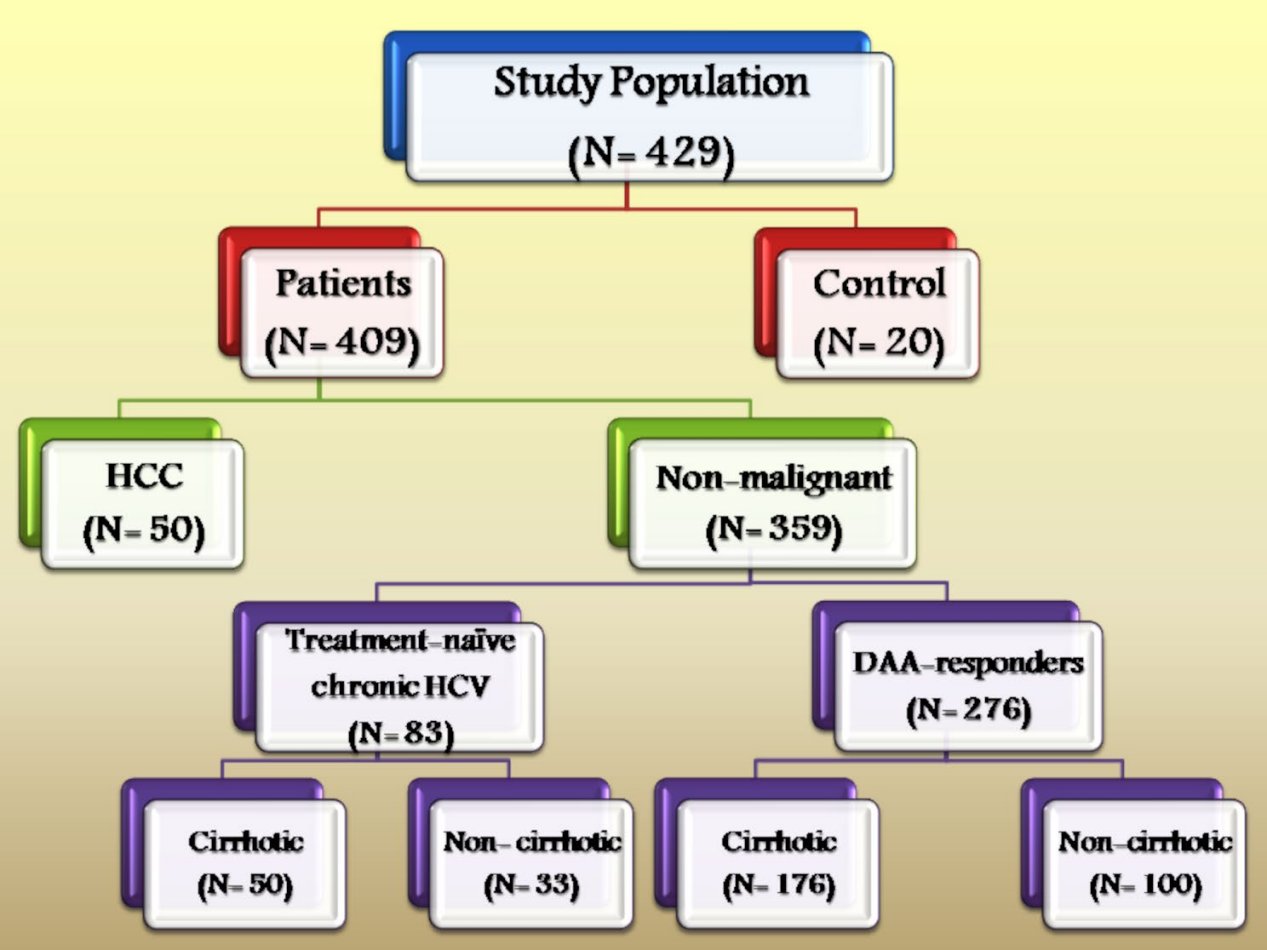
Flow chart of the study population

The study population included six independent subgroups: (1) a *control group* that included healthy patients with no history of liver disease, (2) *cirrhotic DAA-SVR* HCV patients, (3) non-cirrhotic *DAA-SVR* HCV patients, (4) cirrhotic treatment-naïve HCV patients, (5) non-cirrhotic treatment-naïve HCV patients, and (6) anticancer treatment-naïve HCV-related HCC patients. Blood samples (3–5 mL) were collected from patients in different groups that were clinically examined at the *Viral Diseases Outpatient Clinic*, *El-Gomhouria Teaching Hospital***(Cairo**, **Egypt)**, during the period of 2017–2023, which also encompasses the Egyptian National Campaign for HCV *Screening/Treatment (100 million seha*) period (2018–2019). Demographic, clinical, and laboratory data were recorded for each patient using a dedicated electronic *case report form* throughout the treatment period. The primary DAA regimen included a 12-week period of sofosbuvir with or without ribavirin, as indicated for each patient. After completion of the treatment protocol, the viral load was quantified using quantitative PCR (qPCR) with a limit of detection of 21 IU/mL, and sustained virologic response (SVR) was defined as an undetectable serum level of viral RNA three months (minimum) after DAA therapy termination. A flowchart of the study population is shown in Fig. 1.

### Clinical assessment

Liver cirrhosis was determined by biochemical and/or imaging, indicative of clinically significant portal hypertension (i.e., platelet count ≤ 150/mL with splenomegaly and/or esophageal and/or gastric varices), Transient Elastography (liver stiffness measurement [LSM] > 12.5 kPa) [32], or liver biopsy (Metavir score ≥ 4 or Ishak score ≥ 6). Fibrosis (FIB-4) indices were calculated following **Naga et al.** [33] according to the following equation:

$$\displaylines{\:{\text{FIB}} - 4 = {\text{Age}}\:([{\text{yr}}\left] {\:{\text{x}}\:{\text{AST}}\:} \right[{\text{U}}/{\text{L}}])\:/\:(({\text{PLT}}\:[{10^9}/{\text{L}}])\: \cr {\text{x}}\:({\text{ALT}}\:[{\text{U}}/{\text{L}}])(1/2)) \cr}$$

A cutoff value of < 1.45 excluded advanced fibrosis, and values ≥ 3.25 were assigned for marked fibrosis. HCC was diagnosed using contrast-enhanced magnetic resonance imaging (MRI) and computed tomography (CT) according to the American Association for the Study of Liver Disease or European Association for the Study of Liver Disease (AASLD/EASL) guidelines [34, 35]. The Child‒Pugh score was used to determine the functional class of patients with cirrhosis on the basis of prothrombin time, serum albumin (ALB) concentration, total bilirubin concentration, and clinical findings of encephalopathy and ascites. The score is typically 5–6 points for Child‒Pugh class A, 7–9 points for Child‒Pugh class B, or 10–15 points for Child‒Pugh class C [35]. Evidence of portal hypertension includes the occurrence of esophageal, gastric, or splenic varices; splenomegaly; and portal hypertensive gastropathy. The general clinical condition of the HCC patients was assessed according to the Eastern Cooperative Oncology Group (ECOG) Performance score, and the tumor status was evaluated using Barcelona Clinic Liver Cancer (BCLC) staging [36].

### Inclusion and exclusion criteria

The inclusion criteria were cirrhotic/non-cirrhotic treatment-naïve HCV and SVR patients, anticancer treatment-naïve HCV-related HCC patients, or any patients who underwent pathological evaluation in the last 10 years, including primary HCC and metastatic patients, before the primary site was discovered. Patients classified as Child‒Pugh class A or B with compensated liver disease or those with a history of decompensation but showing a stable clinical condition with compensation within a preceding period of 12 months were also included. The exclusion criteria were pregnancy, HBV or HIV co-infection, DAA treatment failure, history of HCC or unclassified nodular lesions, pediatric HCC patients, non-hepatic malignancies, active gastrointestinal bleeding, patients who received neoadjuvant chemotherapy, microwave or radiofrequency ablation, Sorafenib prior to surgery, and patients who underwent or were on the waiting list for liver transplantation.The exclusion criteria for healthy controls included hypertension, diabetes mellitus, autoimmune disorders that might interfere with the study results.

### Biochemical and immunological assessments

The blood samples collected from each patient were equally divided, and half of the samples were used for CBC profiling **(DxH 900**, **Beckman Coulter**, **USA)**. PBMCs were isolated via density gradient centrifugation over a Ficoll^®^ Paque Plus gradient **(Sigma Aldrich-USA)** according to the manufacturer’s protocol. The other half was centrifuged at 3000*x*g for 10 min (RT) to collect plasma samples. Liver alanine aminotransferase (ALT), aspartate aminotransferase (AST), total bilirubin (BiL), and albumin (ALB) were biochemically assessed in plasma by corresponding kits from **Biodiagnostic (Cairo**, **Egypt)** according to the manufacturer’s instructions. The levels of peripheral alpha-fetoprotein (AFP), soluble cluster of differentiation (sCD)163, and interleukins 10 and 12 (IL-10 and IL-12) were estimated by corresponding human ELISA kits from **SunLong Biotech (China)** specific for each marker. The absorbance was measured at 450 nm with reference to 630 nm, and the cutoff value for a positive response was determined according to the minimum detection limit of each kit. The samples were assayed in triplicate, and the results were expressed as the means ± SDs.

### Molecular studies

The expression profiles of the *Cldn*1 and *TGF-β* genes were quantified in isolated PBMCs relative to that of the housekeeping gene GAPDH. Total RNA was extracted from PBMCs using an RNeasy kit from **Thermo Fisher Scientific (USA)** according to the manufacturer’s instructions, and the purified RNA was used for cDNA synthesis using iScript™ RT Supermix **(Bio-Rad**, **USA)**. The reaction mixture included 100 ng of purified RNA, 4 µl of 5X iScript™ Supermix, and each primer at a final concentration of 10 µM (Supplementary Table 1), and the final reaction volume was 20 µl. The thermal cycling program included a 5-min round of priming at 25 °C, followed by reverse transcription for 20 min at 46 °C, and a final round of enzyme inactivation at 95 °C for 1 min. Quantitative PCR (qPCR) was performed using a Rotor-Gene^®^ Q real-time cycler **(QIAGEN GmbH**, **Hilden**, **Germany)**, which included 100 ng of cDNA template, 100 nM final concentration of each primer, and 25 µl of SYBR^®^ Green Master Mix **(Thermo Fisher Scientific**, **USA)**. The reaction volume was adjusted to 50 µl using Q-water. The cycling program was initiated with an activation step at 50 °C for 2 min, followed by a predenaturation step at 95 °C for 10 min; 40 cycles of denaturation for 15 s at 95 °C and annealing for 1 min at 60 °C; and dissociation of the melting curve step, which included 60 s at 95 °C, 15 s at 60 °C, and 15 s at 95 °C. The melting curve was analyzed to confirm specific amplification without primer dimers. The data were analyzed using the comparative threshold cycle method (ΔΔCt), and the geometric mean of GAPDH was used for normalization. The relative expression of *Cldn-1* and *TGF-β* in different patient groups is presented as the fold change relative to the baseline expression in healthy controls (baseline). The samples were assayed in triplicate, and the results are expressed as the means ± SDs.

### Statistical analysis

The sample size was calculated using G-Power software version 3.1.9.7 **(Fraz Faul**, **Germany)**. The Priori calculation of the AST/ALT ratio in the six independent groups included in the study by F-test (ANOVA: fixed effect, omnibus, one way) revealed an actual power of 0.9552, with a total sample size of 234 required to reject the null hypothesis and achieve an effect size (f) of 0.25 and a study power of 95% (1-β error probe); however, the sample size in this study was 429. This number was further assessed using a continuity-corrected squared Fisher’s exact test, with a probability of type I error (α error = 0.05) and power = 95%, as shown in Supplementary Fig. S1. The data were analyzed using GraphPad Prism 9.0.0, where numerical data are presented as the median (min‒max) ± standard error (SE), and qualitative data are presented as numbers and percentages. Multivariate analysis was performed using a logistic regression model to test for the independent predictive effect of statistically significant variables at the multivariate level by calculating the odds ratio and its 95% confidence interval. The sensitivity, specificity, positive predictive value, negative predictive value, and total accuracy were calculated with 95% confidence intervals using receiver operating characteristic (ROC) analysis, and the calculated cutoff values were further validated via Fisher’s exact test. Correlation analysis was performed via Spearman’s correlation matrix. All tests were two-tailed, and *P* values ≤ 0.05 indicated statistical significance.

## Results

### General demographic and clinicopathological criteria of the study population

The study population included several independent cohorts; therefore, each subgroup included a different number of participants. As summarized in Table 1.A, the HCC group had a male predominance, unlike the non-malignant group, which had a higher percentage of female patients, despite the absence of a significant difference. The median age in all patient groups was significantly higher than that in the control group (*P* < 0.0001), whereas the median age in the non-malignant group was significantly lower than that in the HCC cohort (*P* = 0.034–<0.0001). A comparison of the median age of non-/cirrhotic chronic HCV patients with each other and with the corresponding populations of SVRs revealed non-significant differences; however, non-cirrhotic SVRs were younger than cirrhotic SVRs (*P* = 0.0077). Compared with the control group, the largest difference in the median age was observed in the HCC group, followed by the cirrhotic SVRs group (21.41 and 14.54 years, respectively; *P* < 0.0001/each), whereas the non- cirrhotic SVRs had the lowest median age relative to the HCC group (11.51 years, *P* < 0.0001). The general clinical assessment of the patient groups revealed that hypertension and diabetes mellitus were the most common morbidities among the different subgroups of the study population, with HCC patients showing the highest frequency of both hypertension and diabetes mellitus (66% [*P* < 0.0001] and 38% [*P* = 0.0026], respectively), whereas chronic kidney disease (CKD) was the least frequent morbidity, with no significant differences among the patient groups.

**Table 1.A:** Demographic and clinical characteristics of the study cohort (*n* = 429)

Variable	Control (*n* = 20)	Patients (*n* = 409)					*P*-value
		HCC (*n* = 50)	Chronic HCV (*n* = 83)		DAA-Responders (*n* = 276)		
			Cirrhotic	Non-cirrhotic	Cirrhotic	Non-cirrhotic	
Gender (% [*n*/total])							
*Male*	50 (10/20)	62 (31/50)	40 (20/50)	48.48 (16/33)	49.43 (87/176)	45 (45/100)	0.3008
*Female*	50 (10/20)	38 (19/50)	60 (30/50)	51.52 (17/33)	50.57 (89/176)	55 (55/100)	
Age (Years)	36 (23–51) ± 2.263	60 (35–75) ± 1.45	51 (35–65) ± 1.774	48 (40–66) ± 1.167	55 (28–75) ± 0.852	47 (23–66) ± 1.142	< 0.0001
Clinical Assessment (% [*n*/total])							
Hypertension	-	66 (33/50)	48 (24/50)	36.36 (12/33)	45.45 (80/176)	32 (32/100)	0.0026
DM		38 (19/50)	2 (1/50)	3.03 (1/33)	1.14 (2/176)	2 (2/100)	< 0.0001
CDK		0 (0/50)	2 (1/50)	3.03 (1/33)	1.7 (3/176)	2 (2/100)	0.8637

*Abbreviations*: CDK: chronic kidney disease; DM: diabetes mellitus; HCC: hepatocellular carcinoma; DAA: direct-acting antivirals

All data are expressed as median (min-max) ± SE

The hematological profile of the HCC patients was significantly altered compared with that of both the control and non-malignant patients, who had lower counts of red blood corpuscles (RBCs) (*P* = 0.001 and 0.0008–<0.0001) and platelets (PLT) (*P* = 0.0008 and < 0.0001) but no significant difference compared with healthy controls and higher international normalized ratios (INRs) than the non-cirrhotic HCV patients and cirrhotic responders (*P* < 0.0001 and 0.0007, respectively), but no difference was observed compared with the cirrhotic HCV patients and non-cirrhotic responders (Table 1.B). Similarly, the non-malignant patients had higher INRs than the healthy controls (*P* < 0.0001), except for the non-cirrhotic HCV patients, whose INRs were not significantly different. The same was observed for the hemoglobin levels and white blood cell (WBC) counts, which did not differ among all the groups. A comparison of the hematological profiles of non-/cirrhotic patients revealed a significant reduction in the PLT count and higher INRs in cirrhotic patients in both the chronic HCV and SVR groups compared with those in the non-cirrhotic groups (*P* < 0.0001 for both), and in cirrhotic SVRs compared with those in the other non-malignant groups (*P* = 0.01–<0.0001). Moreover, no significant differences were found in RBC or platelet counts between the non-/cirrhotic chronic HCV and the SVR groups.

**Table 1.B:** Hematological profile, liver damage markers, and HCC characteristics assessed in the study cohort (*n* = 429)

Variable	Control (*n* = 20)	Patients (*n* = 409)					*P*-value
		HCC (*n* = 50)	Chronic HCV (*n* = 83)		DAA-Responders (*n* = 276)		
			Cirrhotic	Non-cirrhotic	Cirrhotic	Non-cirrhotic	
Hematological profile							
Hb (g/dL)	13.5 (10.9–15.7) ± 0.301	12.5 (7.3 − 16.5) ± 0.3	12.7 (11.3–15.5) ± 0.302	13 (9.5–15.8) ± 0.19	12.4 (9.5–16.6) ± 0.28	12.7 (9.5–16.6) ± 0.39	0.1191
RBCs (×10^6^/µL)	4.73 (3.97– 5.61) ± 0.09	3.8 (3.2–4.7) ± 0.114	4.53 (3.22– 5.34) ± 0.09	4.42 (3.22– 5.34) ± 0.14	4.74 (3.31–6.2) ± 0.097	4.7 (3.31– 6.2) ± 0.16	< 0.0001
WBCs (×10^3^/µL)	6.3 (3.4–11) ± 0.574	4.55 (1.9 − 12.83) ± 0.417	6.0 (3.6–14.6) ± 0.574	6.5 (3.9–9.1) ± 0.73	6.3 (3.9–14.6) ± 0.59	6.4 (3.6–7.9) ± 0.29	0.0789
PLT (×10^3^/ µL)	270 (161– 370) ± 15.01	168.5 (40– 459) ± 15.2	271 (198 − 560) ± 8.6	285 (198 − 560) ± 12.5	255 (97–422) ± 5.66	308 (187 − 531) ± 6.22	< 0.0001
INR	1.0 (0.9–1.2) ± 0.019	1.3 (0.8–1.69) ± 0.03	1.2 (1.0–1.4) ± 0.023	1.0 (0.9–1.3) ± 0.018	1.27 (0.96 − 1.62) ± 0.01	1.197 (0.91–1.37) ± 0.011	< 0.0001
ALT (IU/L)	24.75 (17.8–34.6) ± 1.357	43.67 (13– 139) ± 3.34	40.31 (11– 74) ± 2.283	34.59 (14– 61) ± 3.178	33 (7.5– 62) ± 0.86	29 (11–49) ± 0.891	< 0.0001
AST (IU/L)	19.9 (13–36.5) ± 1.72	56.81 (14–214) ± 6.38	40 (12–81) ± 2.94	33 (10–69) ± 2.74	38.65 (10 − 78) ± 0.96	22 (9–45) ± 0.735	< 0.0001
AST/ALT	0.8 (0.571–0.96) ± 0.028	1.33 (0.44–4.73) ± 0.12	1.18 (1.04–1.8) ± 0.032	0.77 (0.47–0.97) ± 0.022	1.17 (0.86–3.4) ± 0.018	0.82 (0.37– 1) ± 0.014	< 0.0001
T. BiL (mg/dL)	0.57 (0.5–0.86) ± 0.0234	1.28 (0.33– 13.1) ± 0.26	0.725 (0.4–1.2) ± 0.041	0.69 (0.3–1.11) ± 0.05	1.0 (0.19– 2.17) ± 0.03	0.7 (0.1–1.3) ± 0.028	< 0.0001
Treatment	-	DAA	Naïve		DAA		NA
Log_10_ viral load (IU/mL)	-	SVR	5.967 (4.476 − 7.027) ± 0.0971	5.88 (4.176 − 7.0) ± 0.118	SVR		0.3244
Liver Damage Markers							
ALB (g/dL)	4.9 (4.4–5.0) ± 0.046	3.7 (2.5–4.7) ± 0.072	3.9 (3.22–5.3) ± 0.077	4.5 (3.4–5.7) ± 0.078	4.42 (3.6–5.8) ± 0.046	4.68 (3.5–6.23) ± 0.03	< 0.0001
AFP (ng/mL)	0.493 (0.05 − 2.16) ± 0.123	39.8 (0.7–60,500) ± 1621	3.42 (1.05 − 11.32) ± 0.43	1.8 (0.251–5.04) ± 0.24	2.94 (0.7 − 10.18) ± 0.86	0.48 (0.1–3.89) ± 0.14	< 0.0001
FIB-4	0.46 (0.19–0.55) ± 0.014	2.63 (0.46 − 13.15) ± 0.54	1.5 (0.7–2.6) ± 0.071	0.8 (0.5– 1.42) ± 0.042	1.38 (0.54–4.7) ± 0.054	0.64 (0.19–1.21) ± 0.022	< 0.0001
Child-Pugh class and score							
A5	-	52 (26/50)	95.18 (79/83)	-	99.28 (274/276)	-	< 0.0001
A6		22 (11/50)	4.82 (4/83)		0.72 (2/276)		
B7		26 (13/50)	0.0 (0/83)		0.0 (0/276)		
HCC Characteristics							
BCLC tumor staging (% [n/total])							
A	-	10 (5/50)	-				NA*
B		20 (10/50)					
C		70 (35/50)					
ECOG score (% [n/total])							
0	-	76 (38/50)	-				NA
1		14 (7/50)					
2		4 (2/50)					
3		6 (3/50)					
PVT (% [n/total])							
*Yes*	-	52 (26/50)	-				NA
*No*		48 (24/50)					
LN metastasis (% [n/total])							
*Yes*	-	32 (16/50)	-				NA
*No*		68 (34/50)					
Distant metastasis (% [n/total])							
*Yes*	-	22 (11/50)	-				NA
*No*		78 (39/50)					
Number of tumors (% [n/total])							
Single	-	50 (25/50)	-				NA
2–3		12 (6/50)					
Multiple		38 (19/50)					
Tumor site (% [n/total])							
Left	-	14 (7/50)	-				NA
Right		48 (24/50)					
Bipolar		38 (19/50)					
Maximum tumor diameter (cm) (% [n/total])							
< 3	-	20 (10/50)	-				NA
3–10		50 (25/50)					
> 10		30 (15/50)					
Underlying Morbidity							
Ascites	-	22 (11/50)	-				NA
Splenomegaly							
Mild-moderate		56 (28/50)					
Marked		44 (22/50)					

*Abbreviations*: AFP: alpha fetoprotein; ALB: albumin; ALT: alanine aminotransferase; AST: aspartate aminotransferase; BCLC: Barcelona Clinic Liver Cancer; DAA: direct-acting antivirals; ECOG: Eastern Cooperative Oncology Group performance score; Hb: hemoglobin; HCC: hepatocellular carcinoma; INR: international normalized ratio; LN: lymph nodes; PLT: platelets; PVT: portal vein thrombosis; RBCs: red blood corpuscles; T. BiL: total bilirubin; WBCs: white blood cells

*NA: not applicable

All data are expressed as median (min-max) ± SE

In terms of blood biochemistry, the median levels of ALT did not differ between the cirrhotic SVR and non-cirrhotic chronic HCV/SVR cohorts, and between the cirrhotic SVR and control cohorts, but the AST levels and the AST/ALT ratio were significantly higher in the cirrhotic patients in both non-malignant groups (*P* = 0.0179, 0.0051, and 0.0015, < 0.0001 for chronic HCV patients and SVRs, respectively), while there was no difference among the non-cirrhotic patients. The median levels of both transaminases and the AST/ALT ratio were significantly elevated in the HCC patients compared with the healthy controls (*P* < 0.0001), the SVRs (*P* < 0.0001/all), and the chronic HCV patients (*P* = 0.0383 [ALT], < 0.0001 [AST and AST/ALT]). Both transaminases did not differ between the non-/cirrhotic chronic HCV group and the SVR group; however, the non-cirrhotic SVRs had lower ALT levels (*P* = 0.0032) than did the cirrhotic HCV patients and lower AST levels than did the cirrhotic HCV and SVRs (*P* = 0.0003 and *P* < 0.0001). The median AST/ALT ratio was also lower in both chronic HCV and non-cirrhotic SVRs patients than in cirrhotic patients (*P* < 0.00001). The median level of total bilirubin (BiL) was higher in HCC patients than in non-malignant patients (*P* = 0.0063–<0.0001) and controls (*P* = 0.0064), and in cirrhotic SVRs than in non-cirrhotic patients (*P* = 0.0074), while no difference was detected in the non-/cirrhotic groups compared with the control group.

At the time of sample collection, no viral RNA was detected in the HCC or SVR groups, as both achieved SVR after DAA treatment, whereas the viral loads in the cirrhotic and non-cirrhotic HCV patients did not significantly differ. With respect to the severity of liver damage, patients with HCC had significantly higher FIB-4 and Child‒Pugh scores concomitant with higher AFP levels than non-malignant patients did (*P* < 0.0001/each), but they had lower levels of ALB (*P* < 0.0001). Additionally, the chronic HCV patients had significantly higher AFP levels than the SVRs did, and the same was true for the cirrhotic patients in each group compared with the non-cirrhotic patients (*P* < 0.0001 for all). Although no difference in the serum ALB concentration was detected between non-/cirrhotic chronic HCV patients, the cirrhotic SVRs had higher serum ALB concentrations than did the cirrhotic chronic patients (*P* = 0.0053) but lower levels than did the non-cirrhotic SVRs (*P* = 0.0023).

Assessment of the tumor aggressiveness scores among the HCC patients revealed that 35 patients (70%) had BCLC stage C tumors, 10 patients (20%) had stage B tumors, and 5 patients (10%) had stage A tumors. Among these patients, 37 patients (74%) had an Eastern Cooperative Oncology Group (ECOG) score of zero, whereas only 13 patients (16%) had a score of 1. Portal vein thrombosis (PVT) was observed in 26 patients (52%), whereas lymph node and distant metastases were observed in 16 (32%) and 11 (22%) patients, respectively. Twenty-five patients (50%) had a single tumor, 19 had multiple tumors, and 6 had 2–3 tumors. Most tumors were detected in the right lobe (24 patients (48%)), followed by bipolar tumors (19 patients [38%]), and the least frequent tumors were observed in the left lobe (7 patients [14%]). Tumors with a maximum diameter ranging between 3 and 10 cm were observed in 25 patients (50%), whereas those with a maximum tumor diameter (MTD) > 10 cm were observed in 15 patients (30%), and 10 patients (20%) had tumors with an MTD < 3 cm.

### Differential expression of serum sCD163, IL-10 and IL-12 among cirrhotic and non-cirrhotic patients

**Fig. 2:**
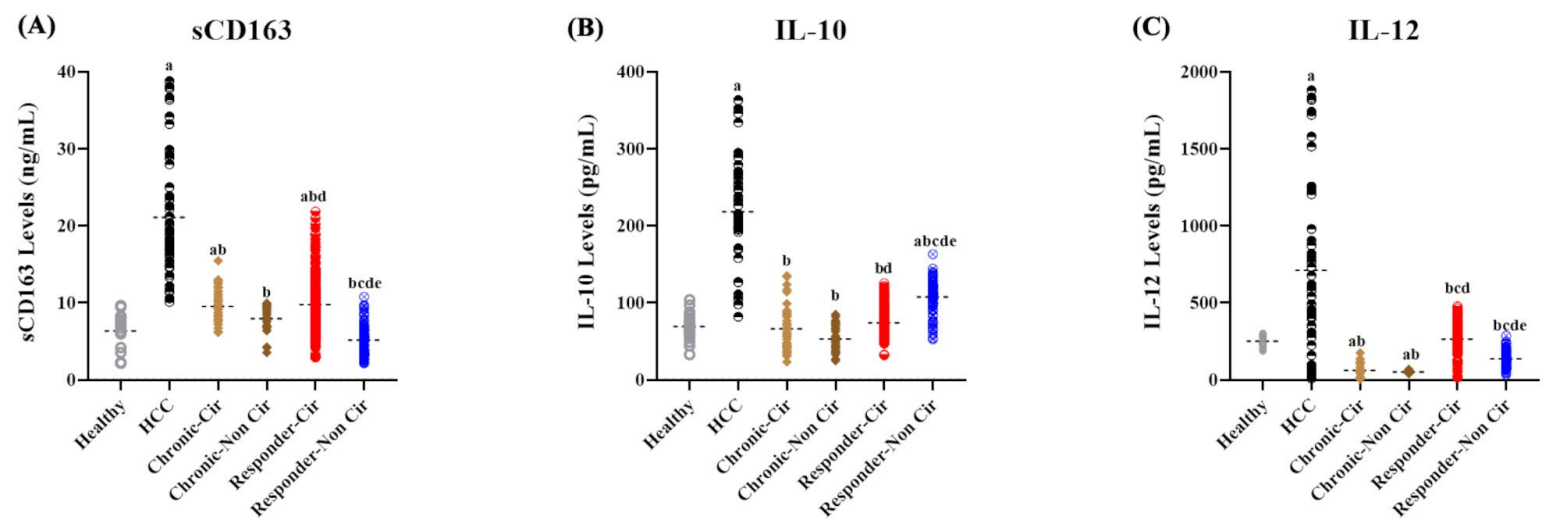
Comparison of the circulating levels of sCD163, IL-10, and IL-12 among different patient groups. The serum sCD163 (**A**), IL-10 (**B**), and IL-12 (**C**) levels were significantly higher in the HCC patient group (*P* < 0.0001) than in the control and non-malignant patients’ groups. No significant difference in the serum levels of the three immune mediators was detected between cirrhotic and non-cirrhotic chronic HCV patients, while the serum levels of sCD163 and IL-12 were higher in cirrhotic SVRs than in non-cirrhotic (*P* < 0.0001 for each), unlike the lower levels of IL-10 in cirrhotic SVRs than in non-cirrhotic patients (*P* < 0.0001)

Compared with those in the control and HCC patient groups, the levels of circulating sCD163, IL-10, and IL-12 were significantly different among non-/cirrhotic patients [F_(5, 422)_ = 27.51, 29.57, and 79.94, respectively, *P* < 0.0001/all] (Fig. 2A-C). The median levels of sCD163 were higher in the HCC patient group than in the control and non-malignant patient groups, with a range of 2.16–4.1-fold (*P* < 0.0001 for all). The levels of sCD163 were 1.5- and 1.54-fold higher in cirrhotic SVRs and chronic HCV patients, respectively, than in controls (*P* = 0.0053 and 0.0358, respectively), while neither of the non-cirrhotic groups were significantly different from the control group. No difference was observed in the serum sCD163 levels between cirrhotic and non-cirrhotic chronic HCV patients, while sCD163 levels were 1.9- and 1.23-fold higher in cirrhotic SVRs and chronic patients, respectively, than in the non-cirrhotic cohort (*P* < 0.0001 and *P* = 0.0085, respectively), with no difference observed relative to those in cirrhotic HCV patients. The median level of IL-10 was the highest in the HCC patients, followed by the non-cirrhotic SVRs, with ranges of 2.06–3.78- and 1.45–2-fold higher than those in the control and other groups (*P* < 0.0001/all), respectively, and none of these values were significantly different from those of the control or each other.

The median serum IL-12 level was higher in patients with HCC than in control and non-malignant patients, with a range of 2.7–13.83-fold (*P* < 0.0001/all), and the level was the lowest in chronic HCV patients, with 54.8–91.34% and 62.3–92.8% reductions in cirrhotic and non-cirrhotic patients, respectively (*P* = 0.009 to < 0.0001). The differences in the serum IL-12 levels between the cirrhotic and non-cirrhotic SVRs were not significant compared with those in the control group; however, the levels in the cirrhotic group were higher than those in the non-cirrhotic group (*P* < 0.0001), and both groups presented 2.21- to 5.14-fold greater IL-12 levels than those in chronic HCV patients (*P* < 0.0001/all).

### Differential transcription of *Cldn1* and *TGF-β* in the PBMCs of cirrhotic and non-cirrhotic patients

**Fig. 3:**
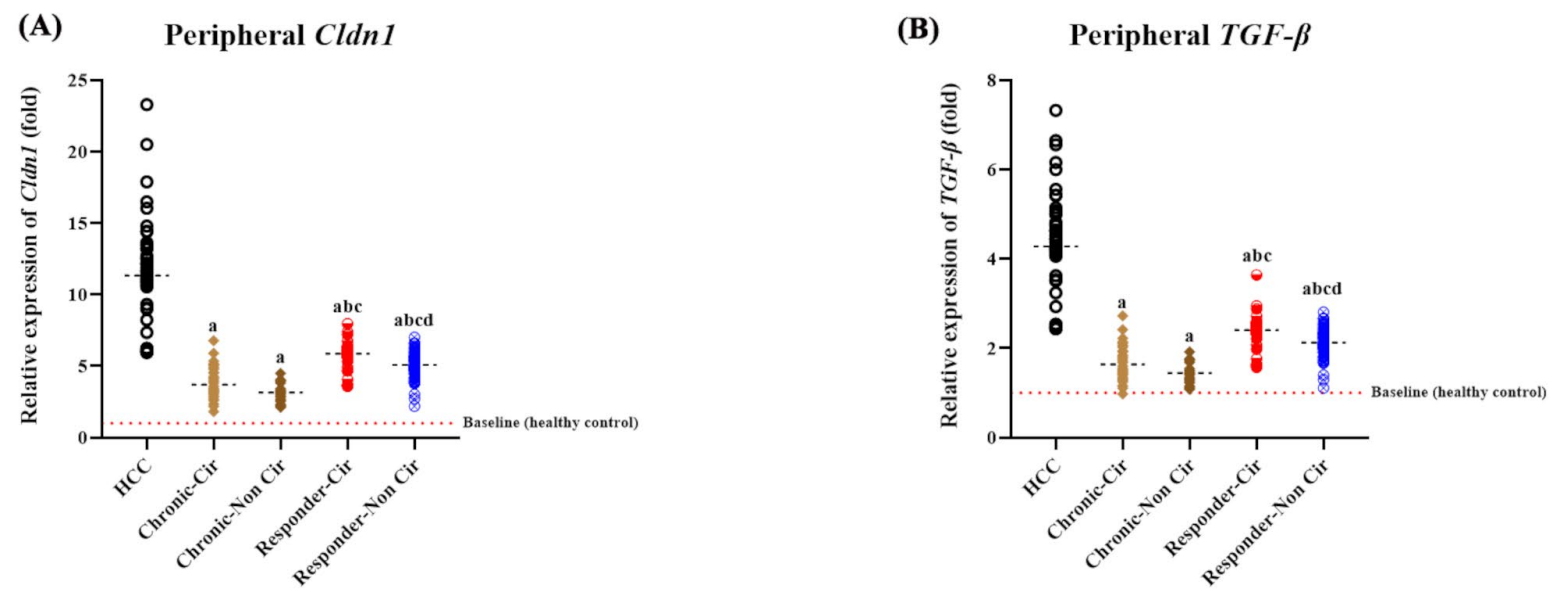
Expression profiles of the *Cldn1* and *TGF-β* genes in the PBMCs of different patient groups relative to the negative control (healthy individuals). The transcriptional levels of peripheral *Cldn1*(**A**) and *TGF-β*(**B**) were significantly higher in the HCC patients and malignant cirrhosis patients than in the non-cirrhotic patients (*P =* 0.0185–<0.0001 and 0.0089–<0.0001, respectively)

Similarly, the transcriptional levels of *Cldn1* and *TGF-β* in PBMCs significantly varied among the different patient groups [F_(4, 404)_ = 54.42 (*P* < 0.0001) and 56.81 (*P* < 0.0001), respectively]. The median fold changes in peripheral *Cldn1* and *TGF-β* in HCC patients were 1.93–3.6- and 1.78–2.95-fold higher, respectively, than those in the other groups (*P* < 0.0001/all). In the chronic HCV patient group, the median fold change values of both genes in cirrhotic patients did not significantly differ from those in the non-cirrhotic group, unlike in the SVR group, in which non-cirrhotic patients presented significantly lower expression levels of both genes than did cirrhotic patients (*P* = 0.0003 and *P* < 0.0001, respectively). Compared with the cirrhotic and non-cirrhotic SVRs, both the non-/cirrhotic HCV patient groups presented 19.64–46.24% and 16.6–39.82% lower expression levels of *Cldn1* (*P* = 0.0185 to < 0.0001) and *TGF-β* (*P* = 0.0089 to < 0.0001), respectively (Fig. 3A, B).

### Clinicopathological features associated with HCC risk in patients with non-malignant cirrhosis

To determine the HCC risk factors in cirrhotic patients, whether treatment-naïve HCV patients or DAA-SVRs, multivariate logistic regression analysis was performed using baseline demographic and clinical data in addition to our putative markers, and the corresponding data from non-cirrhotic patients were used as controls. The analysis revealed that older age, higher Child‒Pugh score, baseline INR, and lower albumin level were more likely to be associated with the risk of HCC (*P* = 0.0156–0.001) (Table 2). Additionally, high expression levels of peripheral *Cldn1* and *TGF-β* and high serum levels of sCD163, IL-10, and IL-12 were significantly associated with the risk of HCC (*P* = 0.022–<0.0001). The highest odds ratios were associated with the expression level of peripheral *TGF-β*, the Child‒Pugh score, and INR (odds ratio: 51.89–57.4), followed by the levels of ALB, serum IL-12, and peripheral *Cldn1* (odds ratio: 5.391–15.11).

**Table 2:** Multivariate logistic regression analysis of clinical features predicting HCC (*n* = 409)

Variable	Estimate	SE	95% CI	t-ratio (Z)	*P*-value	OR (95% CI)
Intercept	-37.766	7.843	-53.137 to -22.395	4.917	< 0.0001	-
Gender: Female (*Referent = Male*)	-0.075	0.762	-1.569 to 1.418	0.1026	0.921	0.928 (0.208 to 4.131)
Age (Years)	0.158	0.055	0.049 to 0.267	2.859	0.004	1.171 (1.051 to 1.306)
Child-Pugh score	3.975	1.199	1.626 to 6.324	5.87	0.001	53.254 (5.082 to 558.04)
Peripheral *Cldn1* (mRNA fold change)	1.685	0.737	0.24 to 3.129	2.072	< 0.0001	5.391 (1.271 to 22.861)
Peripheral *TGF-ß* (mRNA fold change)	4.05	1.153	1.79 to 6.31	3.115	< 0.0001	57.4 (5.99 to 550.017)
Serum sCD163 (ng/mL)	12.15	3.589	-6.118 to 20.59	3.386	0.0007	1.601 (1.276 to 2.008)
Serum IL-10 (pg/mL)	10.98	2.415	-6.873 to 16.68	4.548	< 0.0001	1.023 (1.012 to 1.034)
Serum IL-12 (pg/mL)	3.307	1.527	0.5784 to 6.971	2.166	0.0303	9.303 (2.363 to 47.05)
AFP (ng/mL)	5.777	1.25	3.292 to 9.319	3.783	0.0002	2.22 (1.361 to 3.62)
ALT (IU/L)	0.035	0.06	-0.083 to 0.153	0.4612	0.6446	0.9828 (0.917 to 1.059)
AST (IU/L)	-0.059	0.04	-0.137 to 0.018	0.5298	0.5962	1.022 (0.9408 to 1.091)
Total BiL (mg/dL)	0.517	1.278	-1.988 to 3.022	0.7256	0.4681	0.5548 (0.1121 to 2.130)
ALB (g/dL)	-2.375	0.989	-4.313 to -0.437	2.43	0.0156	15.11 (4.969 to 56.33)
FIB-4	0.898	0.808	-0.686 to 2.482	1.656	0.0985	2.455 (0.504 to 11.963)
PLT (×10^3^/µL)	0.012	0.007	-0.002 to 0.025	0.2971	0.7666	1.012 (0.998 to 1.025)
INR	9.745	1.502	2.882to 16.608	4.227	0.003	51.89 (3.797 to 814.5)

### Prognostic capacity of sCD163, IL-10, IL-12, and peripheral *Cldn1* and *TGF-β* expression in patients with non-malignant cirrhosis

**Fig. 4:**
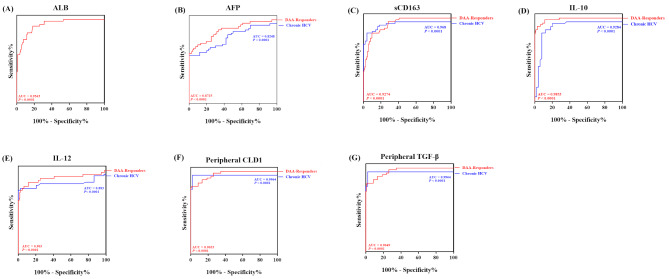
Determination of the ideal cutoff for HCC prediction in patients with non-malignant cirrhosis. AUROC analysis of the serum sCD163 (**C**), IL10 (**D**), and IL-12 (**E**) levels and the mRNA folds of peripheral *Cldn1* (**F**) and *TGF-β*(**G**) revealed a significant AUC value range of 0.883–0.9969, with sensitivity and specificity values ranging from 82 to 100% and 86–98%, respectively, and a considerable CI range, surpassing those of AFP (**B**) and ALB (**A**) in chronic HCV patients who had non-significant AUC values (0.62); therefore, these patients were excluded

Based on the results of the multivariate regression test, variables suggested to be significantly associated with HCC development were further analyzed for significant AUROC values and appropriate thresholds to evaluate their ability to predict HCC development. As shown in Fig. 4 and Table 3, the predicted HCC risk factors had significant areas under the curve (AUCs) (*P* = 0.0007–<0.0001), with AUC values ranging from 0.7708 to 0.9969 and considerable values of sensitivity, specificity, and confidence intervals. Some of the HCC predictors, including age, INR, Child‒Pugh score, and serum ALB concentration in chronic HCV patients, did not have significant enough area under the curve (AUC) values or had a limited range of sensitivity and specificity of the ROC curve and were therefore excluded. Compared with those of ALB (Fig. 4A) and AFP (Fig. 4B), i.e., the gold standard for HCC, the serum levels of sCD163 (Fig. 4C), IL-10 (Fig. 4D), and IL-12 (Fig. 4E) and the mRNA folds of both *Cldn-1* and *TGF-β* (Fig. 4F, G) presented larger AUROCs, with sensitivity and specificity ranges of 82–100% and 86–98%, respectively, and considerable 95% confidence intervals for HCC prediction in non-malignant cirrhotic patients.

**Table 3:** Determination of predictive thresholds of HCC prognostic biomarkers in cirrhotic patients

Variable	Patient group	AUROC Test						Fisher’s Exact Test					
		AUC	SE	95% CI	Cut-off	Sensitivity% (95%CI)	Specificity% (95%CI)	Sensitivity% (95%CI)	Specificity% (95%CI)	PPV% (95%CI)	NPV% (95%CI)	Relative risk (95% CI)	Odds ratio (95% CI)
ALB (g/dL)	*DAA-Responders*	0.9363	0.0173	0.9023 to 0.9702	< 4.23	84 (71.49 to 91.66)	88.07 (82.45 to 92.06)	94.67 (90.19 to 97.17)	71.93 (59.17 to 81.92)	90.91 (85.74 to 94.33)	82 (69.2 to 90.23)	5.051 (2.945 to 9.32)	45.56 (18.69 to 101.8)
AFP (ng/mL)	*Chronic HCV*	0.8248	0.0139	0.9451 to 0.9997	> 5.61	72 (58.33 to 82.53)	70 (56.25 to 80.9)	83.33 (70.42 to 91.3)	80.77 (68.1 to 89.2)	80 (66.96 to 88.76)	84 (71.49 to 91.66)	5.0 (2.746 to 9.715)	21.0 (7.235 to 52.21)
	*DAA-Responders*	0.8725	0.0322	0.8095 to 0.9356	> 5.86	70 (56.25 to 80.9)	90.23 (84.91 to 88.28)	94.12 (89.51 to 96.77)	71.43 (58.52 to 81.58)	90.91 (85.74 to 94.33)	80 (66.96 to 88.76)	4.545 (2.745 to 8.099)	40.0 (16.96 to 98.21)
sCD163 (ng/mL)	*Chronic HCV*	0.968	0.0139	0.9407 to 0.9953	> 11.53	90 (78.64 to 95.65)	86 (73.81 to 93.05)	88 (70.04 to 95.83)	92.16 (81.5 to 96.91)	84.62 (66.47 to 93.85)	94 (83.78 to 98.36)	14.1 (5.126 to 41.44)	86.17 (16.02 to 323.9)
	*DAA-Responders*	0.9274	0.018	0.8922 to 0.9626	> 14.48	82 (69.2 to 90.23)	90.34 (85.08 to 93.88)	97.14 (93.49 to 98.77)	88.24 (76.62 to 94.49)	96.59 (92.76 to 98.43)	90 (78.64 to 95.65)	9.659 (4.519 to 22.23)	255 (73.11 to 854.6)
IL-10 (pg/mL)	*Chronic HCV*	0.9284	0.0291	0.8713 to 0.9855	> 146.5	86 (73.81 to 93.05)	92 (81.16 to 96.85)	85.71 (68.51 to 94.3)	95.83 (86.02 to 99.26)	92.31 (75.86 to 98.63)	92 (81.16 to 96.85)	11.54 (4.851 to 29.41)	138 (24.46 to 648.7)
	*DAA-Responders*	0.9853	0.0071	0.9715 to 0.9992	> 112.1	90 (78.64 to 95.65)	93.75 (89.16 to 96.47)	97.65 (94.11 to 99.08)	82.14 (70.16 to 90)	94.32 (89.86 to 96.88)	92 (81.16 to 96.85)	11.79 (5.002 to 29.91)	190.9 (58.92 to 536.9)
IL-12 (pg/mL)	*Chronic HCV*	0.883	0.0406	0.8035 to 0.9625	> 148	82 (69.2 to 90.23)	97.73 (88.19 to 99.88)	93.88 (83.48 to 97.9)	92.16 (81.5 to 96.91)	92 (81.16 to 96.85)	94 (83.78 to 98.36)	15.33 (5.651 to 44.74)	180.2 (36.42 to 656.7)
	*DAA-Responders*	0.905	0.0368	0.8329 to 0.9772	> 351.2	85.71 (72.16 to 93.28)	88.3 (82.63 to 92.3)	97.09 (93.38 to 98.75)	83.33 (71.26 to 90.98)	94.89 (90.57 to 97.29)	90 (78.64 to 95.65)	9.489 (4.438 to 21.84)	167 (50.23 to 456.8)
*Cldn1* (fold)	*Chronic HCV*	0.9964	0.0039	0.9888 to 1.000	> 5.96	98 (89.5 to 99.9)	98 (89.5 to 99.9)	96 (80.46 to 99.79)	96.08 (86.78 to 99.3)	92.31 (75.86 to 98.63)	98 (89.5 to 99.9)	46.15 (8.759 to 261.4)	588 (57.77 to 6018)
	*DAA-Responders*	0.9653	0.0125	0.9408 to 0.9899	> 6.23	86 (73.81 to 93.05)	91.48 (86.42 to 94.77)	99.41 (96.72 to 99.97)	85.96 (74.68 to 92.71)	95.45 (91.29 to 97.68)	98 (89.5 to 99.9)	47.73 (9.092 to 269.8)	1029 (140.9 to 10411)
*TGF-β* (fold)	*Chronic HCV*	0.9964	0.0039	0.9888 to 1.000	> 2.42	100 (92.87 to 100)	98 (89.5 to 99.9)	89.29 (72.8 to 96.29)	97.92 (89.1 to 99.89)	96.15 (81.11 to 99.8)	94 (83.78 to 98.36)	16.03 (5.905 to 46.74)	391.7 (38.46 to 4037)
	*DAA-Responders*	0.9649	0.0125	0.9403 to 0.9895	> 2.49	90 (78.64 to 95.65)	86.93 (81.15 to 91.13)	98.83 (95.84 to 99.79)	87.27 (75.98 to 93.7)	96.02 (92.02 to 98.06)	96 (86.54 to 99.29)	24.01 (7.131 to 87.01)	579.4 (122.4 to 2527)

**Fig. 5:**
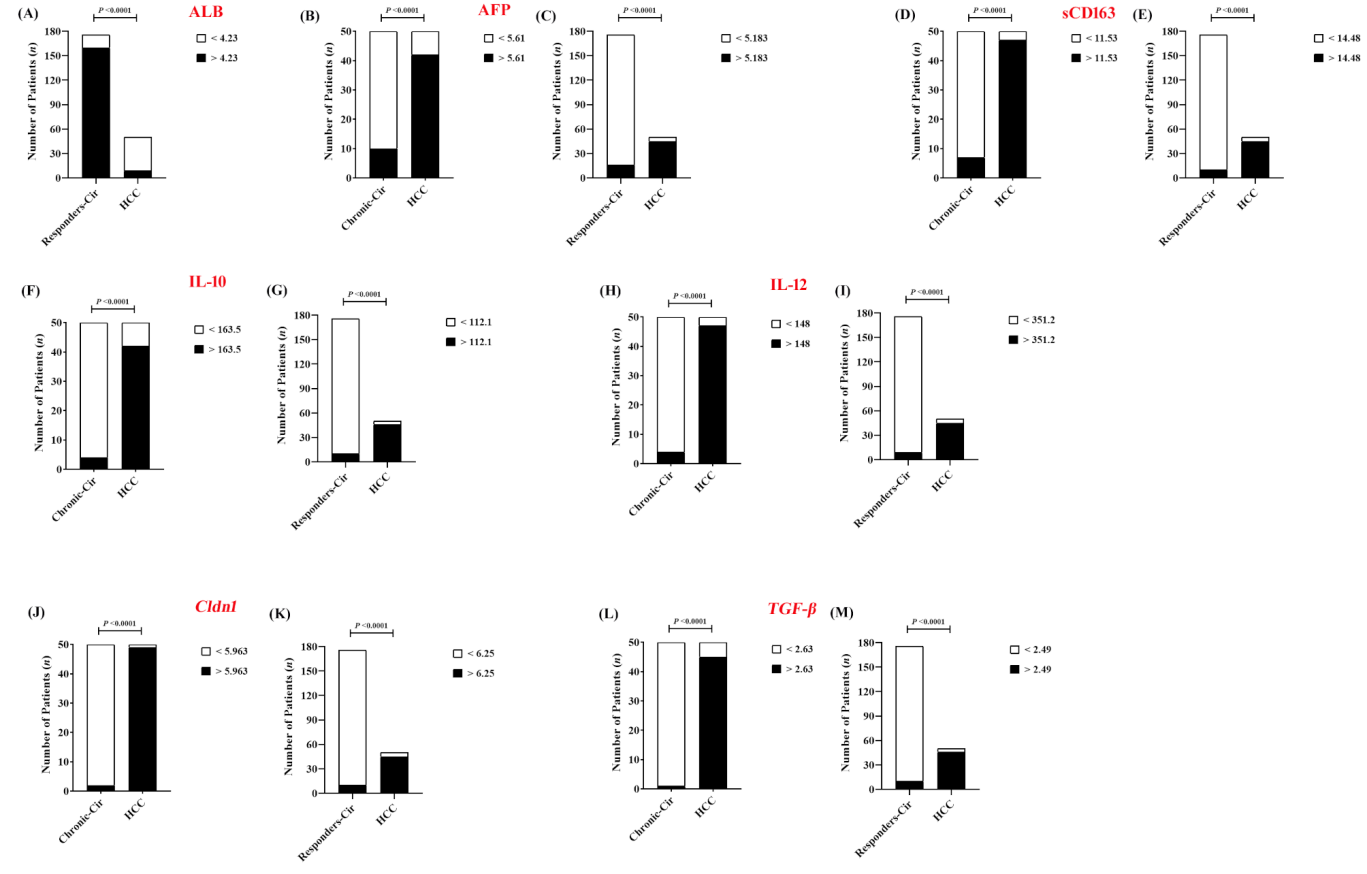
The prognostic performance of the calculated cutoff values was further confirmed by Fisher’s exact test, which revealed a significant predictive power of ALB in SVRs (**A**) and AFP (**B**, **C**), sCD163 (**D**, **E**), IL-10 (**F**, **G**), and IL-12 (**H**, **I**) in the treatment-naïve HCV and SVR cohorts and, similarly, the mRNA folds of *Cldn1*(**J**, **K**) and *TGF-β* in the PBMCs of both cohorts. The sensitivity and specificity values ranged from 88 to 99.41% and 82.14–97.92%, respectively, and the positive/negative predictive values (PPV and NPV) ranged from 84.62 to 98.3% and 92–98%, respectively

The performance of the calculated thresholds was further assessed via Fisher’s exact test, which revealed considerable prognostic capacity of serum AFP (Fig. 5B, C), sCD163 (Fig. 5D, E), IL-10 (Fig. 5F, G), and IL-12 (Fig. 5H, I), as well as peripheral *Cldn1* (Fig. 5J, K) and *TGF-β* (Fig. 5L, M) mRNA folds, with sensitivities and specificities ranging from 88 to 99.41% and 82.14–97.92%, respectively, and 84.62–98.3% and 92–98% positive and negative predictive values, respectively, while serum ALB (Fig. 5A) showed the least predictive power.

### Associations between serum sCD163, IL-10, and IL-12 levels and peripheral *Cldn1* and *TGF-β* expression profiles and clinicopathological features in different patient cohorts

To elucidate their association with pathogenesis, the correlations between putative HCC predictors and other clinical markers were analyzed. In HCC patients, the transcriptional levels of peripheral *Cldn1* and *TGF-β* in PBMCs were strongly positively correlated with each other, and both were inversely correlated with the serum ALB concentration; however, they were positively correlated with ALT, AST, BiL, AFP, and FIB-4 scores and with the serum levels of IL-10, IL-12, and sCD163. The sCD163 level was positively correlated with the IL-10 and IL-12 levels, while the serum IL-10 level was inversely correlated with the Child‒Pugh score but positively correlated with the IL-12 level (Supplementary Table 2). In non-malignant cirrhotic patients, the mRNA folds of peripheral *Cldn1* and *TGF-β* were also positively correlated with each other, and both were positively correlated with AFP, ALB, and the INR; however, no significant correlations were detected with liver transaminases. Moreover, the transcript levels of both genes showed strong positive correlations with the serum levels of IL-12, IL-10, and sCD163. The sCD163 level was inversely correlated with the BiL and ALB levels, as well as the PLT, whereas the serum IL-10 level was positively correlated with the INR. Serum sCD163 and IL-10 levels were positively correlated, and both were positively correlated with IL-12 levels, which in turn were positively correlated with AFP and ALB levels (Supplementary Table 3).

In non-cirrhotic patients, the mRNA fold of peripheral *Cldn1* and *TGF-β* were positively correlated with each other and were positively correlated with AFP, ALT, AST, ALB, INR, serum IL-10, and IL-12. Interestingly, the transcript levels of both genes were inversely correlated with the serum sCD163 level, which was inversely correlated with the IL-10 and IL-12 levels. IL-10 and IL-12 levels showed strong positive correlations with each other and with AFP and the INR, while both were inversely correlated with age and the FIB-4 score. Moreover, the serum sCD163 concentration was inversely correlated with the AFP and INR but positively correlated with the ALT and AST levels, whereas the IL-10 concentration was inversely correlated with the AST level (Supplementary Table 4).

## Discussion

Clinical studies of DAAs in cirrhotic HCV patients performed during the first few years of treatment have generated heated debates about the risk of HCC emergence after patients achieve an SVR [5–7]. Viral elimination with DAA treatment has been postulated to often be accompanied by upregulated expression of immunoregulatory mediators and/or growth factors, particularly vascular endothelial growth factor (VEGF) [33, 37], in addition to certain changes in the immunological milieu, particularly the T-cell-mediated milieu, which might together favor HCC development as a result of reduced antitumor immune surveillance [38]. This issue remains a major concern when addressing the long-term risks of direct-acting antivirals, particularly because the mechanisms underlying oncogenic programming that induce HCC after SVR achieved by DAAs are still obscure. Considering these repercussions, this observational study provides new insights into the early prediction of HCC among cirrhotic HCV patients before and after achieving SVR by DAA therapy and the potential pathways underlying their involvement in hepatocarcinogenesis.

Overall, the clinical assessment of different study groups confirmed the reported hallmarks of liver cirrhosis/carcinogenesis, including male predominance, older age, low platelet count and albumin levels, and higher INR, AFP levels, and AST/ALT ratios in both HCC and cirrhotic non-malignant patients than in non-cirrhotic patients [6–8, 38, 39]. As expected, liver damage was more pronounced among HCC patients in terms of markedly higher FIB-4 and Child‒Pugh scores, in addition to lower albumin levels and platelet counts, than among non-malignant patients, of whom the majority had mild cirrhosis and only a minor fraction had moderate fibrosis. Notably, the AFP levels in SVRs were significantly lower than those in treatment-naïve HCV patients, even those with cirrhosis, in accordance with the previous findings of **Nguyen et al.** [40] and, more recently, **Elbadry et al.**, who assessed the impact of DAAs on the health-related quality of life of Egyptian SVR patients [41]. Despite remaining within normal limits (≤ 10 ng/mL), these findings do not exclude the risk of HCC; in particular, ∼ 30% of patients with HCC have AFP levels within the normal range [16, 17] and further raise questions about the utility of AFP as a gold standard for HCC prognosis in cirrhotic DAA-SVRs with normal serum levels, considering the high AST/ALT ratios, high INR values, and thrombocytopenia, which reflects the need for more specific HCC predictors.

To this end, the performance of soluble CD163 (sCD163) and different cytokines has been assessed in several clinical studies of cirrhotic patients with chronic liver diseases and/or HCC, providing efficient non-invasive markers for HCC risk and/or progression [42–45]. In line with these findings, the circulating levels of sCD163 and IL-10 were higher in HCC patients than in control and non-malignant cohorts, which is consistent with the findings of **Minami et al.** [46], who reported significant upregulation of CD163-expressing tumor-associated macrophages (TAMs) in HCC patients with poorly differentiated tumors and those with tumor sizes ≥ 3.5 cm, and with the findings of **Zhang et al.** [47], who proposed sCD163 as a potential predictor of mortality in patients with decompensated liver cirrhosis. Additionally, the serum IL-10 and IL-12 levels in patients with HCC similarly surpassed those in control and non-malignant patients by up to 3.78- and 13.83-fold, respectively, arguing against the recently reported antitumor role of IL12 [17]. However, our results are in agreement with those of **Shakiba et al.** [48] and **El-Emshaty et al.** [49], who reported significantly upregulated serum levels of IL-10 and IL-12 in patients with chronic liver disease, suggesting that persistent chronic inflammation is associated with HCC risk.

Similarly, the expression of the *Cldn*1 and *TGF-β* genes in the PBMCs of HCC patients was markedly higher than that in the PBMCs of non-malignant patients by up to 3.6- and 2.95-fold, respectively. Although the protumorigenic role of epithelial *Cldn1* expression in hepatocytes has been documented [50, 51], the role of its expression in PBMCs in the context of hepatocarcinogenesis has yet to be elucidated. However, *Cldn1* is the major claudin expressed on M2 macrophages during inflammation and/or carcinogenesis and is prominently expressed in response to TGF-β and IL-10 [21], which explains the increased expression levels of *Cldn1* in HCC patients and the inseparable correlation observed in all study groups. This finding is also reminiscent of the central role of *IL-10 and TGF-β* in liver tumorigenesis, where it has been postulated that the interplay between intrahepatic IL-10 and TGF-β determines fibrogenic severity in chronic HCV patients, which consequently relates to the risk of HCC development/progression [21, 22, 52].

Taken together, these findings are consistent with the results of logistic regression, which revealed a significant association between serum sCD163, IL-10, and IL-12 levels and the transcription signatures of peripheral *Cldn1* and *TGF-β* and a higher risk of HCC. In light of these results, it can be assumed that the co-expression of *Cldn1* and *TGF-β* in the PBMCs of HCC patients under the influence of immunoregulatory mediators, i.e., IL-10 and sCD163, contributes to the perpetuation of the immunosuppressive TME, which directly correlates with tumorigenesis. Considering that logistic regression analysis confirmed the predictive power of *Cldn1* and *TGF-β* mRNA expression in PBMCs from HCC patients, in addition to the reported associations of high serum and/or hepatic expression of sCD163 and IL-10 with poor HCC prognosis [26, 42, 53], the implication of the transcriptional signatures of both genes in hepatocarcinogenesis seems plausible. Although M2 macrophages are prominent TME residents [54], they can also be found in peripheral blood, particularly in inflammatory and/or malignant environments [55, 56], particularly because they originate from peripheral monocytes [57]. This finding provides another mechanism that could underlie the contribution of peripheral Cldn1 to liver tumorigenesis related to leukocyte extravasation. It has been proposed that tight junction proteins (TJPs) expressed on leukocytes can compete with those expressed on endothelial cells (ECs) through their exposed extracellular loops, which opens the epithelial paracellular space in a zipper-like manner and therefore facilitates the infiltration of leukocytes [58]. According to this theory, peripheral *Cldn1* perpetuates tumorigenesis by activating the recruitment of tumor-infiltrating M2 macrophages, which is consistent with the reported role of *Cldn1* in pro-HCC [59].

Based on these data, despite the absence of a significant difference in IL-12 levels in SVRs compared with controls and the lower levels observed in chronic HCV patients, which might be related to the inhibitory effect of the viral core protein on IL-12p40 mRNA synthesis in macrophages [60], the upregulated serum levels of the three immune mediators along with cirrhosis and upregulated expression of the *Cldn1* and *TGF-β* genes are suggestive of potential DAA-mediated activation of HCC-promoting proinflammatory and/or immunoregulatory pathways, considering that all are associated with the risk of HCC according to the logistic regression analysis results. To determine the predictive threshold of HCC risk among these patients, the putative predictors, along with the gold standard, were assessed via AUROC analysis. Except for serum IL-12 in chronic HCV patients who had overall low levels, all putative predictors had AUROC values > 0.9 with considerable CI ranges and sensitivity, specificity, and positive/negative predictive values, unlike albumin and AFP, which demonstrated lower prognostic performance in chronic HCV patients and SVRs, respectively. This suboptimal prognostic capacity could be related to the mild fibrotic grades observed in the majority of cirrhotic patients, which were reflected in the AFP and albumin levels typically associated with the severity of liver damage [17], and therefore showed nearly normal levels of both markers, especially among SVRs.

The pattern of correlations displayed between the new predictors and other clinicopathological markers sheds some light on the possible mechanisms underlying their role in pathogenesis. For example, the positive inter-correlations observed in HCC patients and non-malignant cirrhotic patients between serum sCD163, IL-10, and IL-12 and their strong association with the transcriptional signatures of the peripheral *Cldn1* and *TGF-β* genes support our theory about their role in liver cirrhosis/tumorigenesis in an orchestrated manner. In particular, peripheral *TGF-β* expression levels were strongly correlated with AFP levels in HCC patients and all non-malignant patients. Furthermore, the expression levels of both genes were positively correlated with the FIB-4 score, despite the absence of correlations between the new predictors and any of the HCC hallmarks. However, the negative correlation observed between the serum IL-10 concentration and the Child‒Pugh score might indicate an immune-homeostatic role of the former, where it could act as an “endogenous danger signal” under certain conditions of hyperinflammation, including cancer [61], presumably due to the persistent upregulation of IL-12. Therefore, it tends to promote the immunoregulatory pathways necessary to halt liver damage, which also explains its strong association with the transcription profiles of peripheral *Cldn1*and *TGF-β.* In another context, the inverse correlation observed in non-cirrhotic patients between sCD163 and both interleukins 10 and 12, as well as between peripheral *Cldn1* and *TGF-β* transcription, was not surprising, considering that sCD163 mainly promotes liver cirrhosis by activating hepatic Kupffer cells that consequently overexpress IL-10 [47]. Therefore, in the absence of cirrhosis and associated chronic inflammation mediated by IL-12, an inverse correlation can be expected.

## Conclusion

In light of these findings, serum levels of sCD163, IL-10, and IL-12 and peripheral expression of Cldn1 and TGF-β provide novel non-invasive biomarkers with a significant predictive capacity for HCC risk among patients with mild degrees of liver cirrhosis, circumventing the drawbacks of conventional prognostic markers in patients with normal AFP, albumin, and/or platelet counts. Putative predictors are privileged by maintaining a consistent pattern of association with different clinical markers among different patient cohorts, which confirms their predictive power under different pathological conditions.

### Limitations of the study

This study was limited by the small sample size of patients with chronic HCV, which could be related to the successful national campaign for HCV surveillance and treatment in Egypt. The same applies to patients with *de novo* HCC following DAA therapy who agreed to participate in our study. Further investigations using larger cohorts are needed to validate our results.

## Electronic supplementary material

Below is the link to the electronic supplementary material.

Supplementary Material 1

## Data Availability

No datasets were generated or analysed during the current study.

## Abbreviations

AASLD/EASL: American Association for the Study of the Liver Disease or European Association for the Study of Liver Disease
AFP: Alpha fetoprotein
ALB: Albumin
ALT: Alanine aminotransferase
AST: Aspartate aminotransferase
AUC: Area under curve
BCLC: Barcelona Clinic Liver Cancer
BiL: Bilirubin
CKD: Chronic kidney disease
Cldn: Claudin
CT: Computed tomography
CTLs: Cytotoxic T lymphocytes
DAAs: Direct-acting antivirals
ECOG: Eastern Cooperative Oncology Group performance score
ECs: Endothelial cells
GAPDH: Glyceraldehyde 3-phosphate dehydrogenase
HBV: Hepatitis B virus
HCC: Hepatocellular carcinoma
HCV: Hepatitis C virus
HIV: Human immunodeficiency virus
IL: Interleukin
INR: International normalized ratio
MRI: Magnetic resonance imaging
NAFLD: Non-alcoholic fatty liver disease
NK: Natural killers
PBMCs: Peripheral blood mononuclear cells
PLT: Platelets
PVT: Portal vein thrombosis
RBCs: Red blood corpuscles
ROC: Receiver operating characteristic
sCD163: Soluble cluster of differentiation 163
SVR: Sustained virologic response
TAMs: Tumor-associated macrophages
TGF-β: Transforming growth factor-β
TJPs: Tight junction proteins
TME: Tumor microenvironment
WBCs: White blood cells
