# Correction: Peripheral immune signatures associated with the risk of hepatocarcinogenesis in cirrhotic Egyptian HCV patients before and after treatment with direct-acting antivirals

**DOI:** 10.1186/s12985-024-02598-2

**Published:** 2024-12-12

**Authors:** Reem El-Shenawy, Rehab I. Moustafa, Naiera M. Helmy, Yasmine S. El-Abd, Ashraf A. Tabll, Yasser K. Elesnawy, Heba Shawky

**Affiliations:** 1 Microbial Biotechnology Department, Biotechnology Research Institute, National Research Centre, Dokki, Cairo, 12622 Egypt; 2 School of Pharmacy, Newgiza University (NGU), Newgiza, 12577 Giza Egypt; 3 Egypt Center for Research and Regenerative Medicine (ECRRM), Cairo, Egypt; 4 National Committee for Control of Viral Hepatitis (NCCVH), Ministry of Health and Population, Cairo, Egypt; 5 Therapeutic Chemistry Department, Pharmaceutical Industries and Drug Research Institute, National Research Centre, Dokki, Cairo, 12622 Egypt

**Correction: Virology Journal (2024) 21:293**

10.1186/s12985-024-02551-3

In the online published article 10.1186/s12985-024-02551-3, the sentence beginning “No significant difference was detected” in the Results of abstract section was incorrect. The author would like to correct it.

The sentence in the Results section should be corrected as:

No significant difference was detected in the serum levels of the three markers between cirrhotic and non-cirrhotic patients of chronic HCV, whereas their levels were significantly different between cirrhotic and non-cirrhotic SVRs (*P* < 0.0001).

And, the sentence beginning with “The proposed HCC predictors are” in the Conclusion of the abstract section needs to be replaced with the following text: “Serum sCD163, IL-10, IL-12 and peripheral Cldn1 and TGF-β expression levels represent”.

The original article has been corrected.
