# Supplementary material for: Peripheral immune signatures associated with the risk of hepatocarcinogenesis in cirrhotic Egyptian HCV patients before and after treatment with direct-acting antivirals
Source: Virol J. 2024 Nov 15;21:293. doi: 10.1186/s12985-024-02551-3 (PMC11566392; doi:10.1186/s12985-024-02551-3)
Supplement: Supplementary file 1 — Supplementary Material 1 [file 12985_2024_2551_MOESM1_ESM.docx]

**Peripheral Immune Signatures Associated with the Risk of Hepatocarcinogenesis in Cirrhotic Egyptian HCV Patients Before and After Treatment with Direct-Acting Antivirals**

*Running Title: New Insights for the Early Prediction of HCC*

**Reem M. Elshenawy[
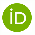
](https://orcid.org/0000-0001-7861-238X), Rehab I. Moustafa[
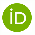
](https://orcid.org/0000-0002-8277-8304), Naiera M. Helmy[
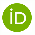
](https://orcid.org/0000-0003-3788-3372), Yasmine S. El-Abd[
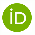
](https://orcid.org/0000-0002-8212-6146), Ashraf A. Tabll[
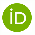
](https://orcid.org/0000-0001-7041-5445), Yasser K. Elesnawy[
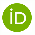
](https://orcid.org/0000-0002-9863-5551), Heba Shawky[
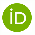
](https://orcid.org/0000-0001-8403-1143)***

**Table of Contents**

[**Supplementary Figures**](#SUPPLEMENTARY_Figures)

[Fig. (S1): Determination of sample size using G-Power software](#SUPPLEMENTARY_Fig_S1) 2

[**Supplementary Tables**](#SUPPLEMENTARY_Tables)

[Supplementary Table 1: Primer sets used for profiling gene expression profiling in PBMCs 3](#SUPPLEMENTARY_Table_1)

[Supplementary Table 2: Correlations between our putative HCC predictors and clinicopathological markers in HCC patients 4](#SUPPLEMENTARY_Table_2)

[Supplementary Table 3: Correlations between our putative HCC predictors and clinicopathological markers in patients with non-malignant cirrhosis 5](#SUPPLEMENTARY_Table_3)

[Supplementary Table 4: Correlations between our putative HCC predictors and clinicopathological markers in non-cirrhotic patients 6](#SUPPLEMENTARY_Table_4)

**
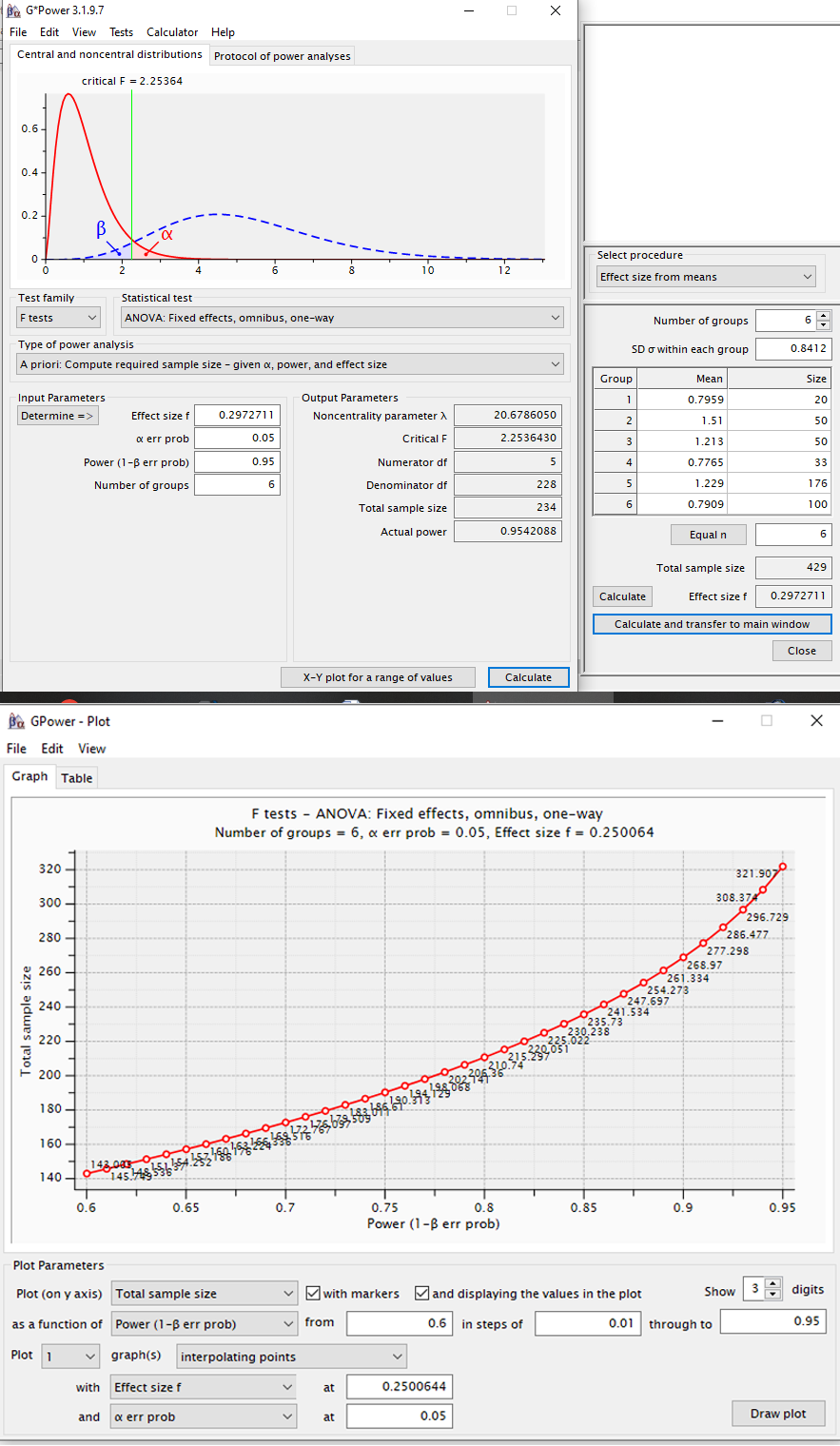
Supplementary Figures**

**Fig. (S1):** Determination of sample size using G-Power software. The Priori test analysis of the AST/ALT ratio in the six independent groups included in the study by F-test (ANOVA: fixed effect, omnibus, one way) revealed an actual power of 0.9552, with a total sample size of 234 required to reject the null hypothesis, and achieve an effect size (f) of 0.25 and a study power of 95% (1-β error probe).

**Supplementary Tables**

**Supplementary Table 1: Primer sets used for gene expression profiling in PBMCs**

| Gene | Primer sequence | *Primer Efficiency |
| --- | --- | --- |
| *Cldn1* | F: 5’-GTCTTTGACTCCTTGCTGAATCTG-3’  R: 5'-CACCTCATCGTCTTCCAAGCAC-3' | 98.84% |
| *TGF-β* | F: 5'- TACCTGAACCCGTGTTGCTCTC-3'  R: 5'- GTTGCTGAGGTATCGCCAGGAA-3' | 109.41% |
| GAPDH | F: 5'- GTCTCCTCTGACTTCAACAGCG-3'  R: 5'- ACCACCCTGTTGCTGTAGCCAA-3' | **-** |

*Primer efficiency was calculated based on the slope of the standard curve established between Ct and log DNA copy number (amplification factor= 2) using the calculator of **Thermo Scientific Web Tools** available from <https://www.thermofisher.com/eg/en/home/brands/thermo-scientific/molecular-biology/molecular-biology-learning-center/molecular-biology-resource-library/thermo-scientific-web-tools/qpcr-efficiency-calculator.html>

**Supplementary Table 2: Correlations between putative HCC predictors and clinicopathological markers in HCC patients (*n*=50)**

|  | Age | Child-Pugh | AFP | ALT | AST | BiL | ALB | FIB-4 | PLT | INR | sCD163 | IL-10 | IL-12 | *Cldn1* | *TGF-β* |
| --- | --- | --- | --- | --- | --- | --- | --- | --- | --- | --- | --- | --- | --- | --- | --- |
| Age | - | *NS | NS | NS | NS | NS | NS | NS | NS | NS | NS | NS | NS | NS | NS |
| Child-Pugh |  | - | NS | rho=0.3058  *P*=0.0308 | NS | NS | NS | NS | NS | NS | NS | rho=-0.2914  *P*=0.0401 | NS | NS | NS |
| AFP |  |  | - | NS | rho=0.497  *P*=0.0002 | rho=0.3721  *P*=0.0078 | NS | rho=0.4251  *P*=0.0021 | NS | NS | NS | NS | NS | rho=0.2848  *P*=0.045 | rho=0.2951  *P*=0.0375 |
| ALT |  |  |  | - | rho=0.7531  *P*<0.0001 | rho=0.403  *P*=0.0037 | rho=-0.3036  *P*=0.0321 | rho=0.3956  *P*=0.0045 | NS | rho=0.3556  *P*=0.0113 | NS | NS | NS | rho=0.4149  *P*=0.0027 | rho=0.4238  *P*=0.0022 |
| AST |  |  |  |  | - | rho=0.5316  *P*<0.0001 | rho=-0.4516  *P*=0.001 | rho=0.734  *P*<0.0001 | rho= -0.3337  *P*=0.0179 | rho=0.5187  *P*=0.0001 | NS | NS | NS | rho=0.538  *P*<0.0001 | rho=0.5405  *P*<0.0001 |
| BiL |  |  |  |  |  | - | rho=-0.403  *P*=0.0037 | rho=0.4201  *P*=0.0024 | rho=-0.2825  *P*=0.0468 | rho=0.5563  *P*<0.0001 | NS | NS | NS | rho=0.3893  *P*=0.0052 | rho=0.3905  *P*=0.005 |
| ALB |  |  |  |  |  |  | - | rho=-0.5436  *P*<0.0001 | rho=0.4605  *P*=0.0008 | rho=-0.5222  *P*=0.0001 | NS | NS | NS | rho=-0.324  *P*=0.0217 | rho=-0.3486  *P*=0.0131 |
| FIB-4 |  |  |  |  |  |  |  | - | rho=-0.7854  *P*<0.0001 | rho=0.5323  *P*<0.0001 | NS | NS | NS | rho=0.2965  *P*=0.0366 | rho=0.3112  *P*=0.0278 |
| PLT |  |  |  |  |  |  |  |  | - | rho=-0.3743  *P*=0.0074 | NS | NS | NS | NS | NS |
| INR |  |  |  |  |  |  |  |  |  | - | NS | NS | NS | NS | NS |
| sCD163 |  |  | | | | | | | | | - | rho=0.5197  *P*<0.0001 | rho=0.9073  *P*<0.0001 | rho=0.4913  *P*=0.0003 | rho=0.482  *P*=0.0004 |
| IL-10 |  |  |  |  |  |  |  |  |  |  |  | - | rho=0.5339  *P*<0.0001 | rho=0.5448  *P*<0.0001 | rho=0.5333  *P*<0.0001 |
| IL-12 |  |  |  |  |  |  |  |  |  |  |  |  | - | rho=0.4751  *P*=0.0005 | rho=0.4608  *P*=0.0008 |
| *Cldn1* |  |  |  |  |  |  |  |  |  |  |  |  |  | - | rho=0.997  *P*<0.0001 |

***NS**: non-significant

**Supplementary Table 3: Correlations between putative HCC predictors and clinicopathological markers in non-malignant cirrhotic patients (*n*=226)**

|  | Age | Child-Pugh | AFP | ALT | AST | BiL | ALB | FIB-4 | PLT | INR | sCD163 | IL-10 | IL-12 | *Cldn1* | *TGF-β* |
| --- | --- | --- | --- | --- | --- | --- | --- | --- | --- | --- | --- | --- | --- | --- | --- |
| Age | - | *NS | NS | rho=-0.2123  *P*=0.0013 | rho=-0.2375  *P*=0.0003 | NS | NS | rho=4286  *P*<0.0001 | NS | NS | NS | NS | NS | NS | NS |
| Child-Pugh |  | - | rho=-0.2088  *P*=0.0016 | NS | NS | NS | NS | NS | NS | NS | NS | NS | NS | NS | NS |
| AFP |  |  | - | rho=-0.1523  *P*=0.0003 | rho=-0.1994  *P*=0.0026 | rho=0.3105  *P*<0.0001 | rho=0.2455  *P*=0.0002 | rho=-0.3017  *P*<0.0001 | rho=0.2722  *P*<0.0001 | rho=0.4417  *P*<0.0001 | NS | NS | rho=4969  *P*<0.0001 | rho=0.4438  *P*<0.0001 | rho=0.4432  *P*<0.0001 |
| ALT |  |  |  | - | rho=0.9154  *P*<0.0001 | rho=0.3608  *P*<0.0001 | rho=0.3419  *P*<0.0001 | rho=0.1437  *P*=0.0308 | NS | rho=-0.2667  *P*<0.0001 | NS | NS | NS | NS | NS |
| AST |  |  |  |  | - | rho=0.3223  *P*<0.0001 | rho=0.3017  *P*<0.0001 | rho=0.2729  *P*<0.0001 | NS | rho=-0.2775  *P*<0.0001 | NS | NS | NS | NS | NS |
| BiL |  |  |  |  |  | - | rho=0.3516  *P*<0.0001 | rho=-0.1755  *P*=0.0082 | rho=0.3692  *P*<0.0001 | NS | rho=-0.2113  *P*=0.0014 | NS | NS | rho=0.3893  *P*=0.0052 | rho=0.3905  *P*=0.005 |
| ALB |  |  |  |  |  |  | - | NS | rho=0.235  *P*=0.0004 | rho=0.1712  *P*=0.0099 | rho=-0.1464  *P*=0.0277 | NS | rho=2336  *P*=0.0006 | rho=0.2475  *P*=0.0002 | rho=-0.2462  *P*=0.0002 |
| FIB-4 |  |  |  |  |  |  |  | - | rho=-0.7224  *P*<0.0001 | NS | NS | NS | NS | NS | NS |
| PLT |  |  |  |  |  |  |  |  | - | NS | rho=-0.2155  *P*=0.0011 | NS | NS | NS | NS |
| INR |  |  |  |  |  |  |  |  |  | - | NS | rho=0.1367  *P*=0.0442 | NS | rho=0.3286  *P*<0.0001 | rho=0.3291  *P*<0.0001 |
| sCD163 |  |  | | | | | | | | | - | rho=0.7476  *P*<0.0001 | rho=0.6605  *P*<0.0001 | rho=0.5221  *P*<0.0001 | rho=0.5206  *P*<0.0001 |
| IL-10 |  |  |  |  |  |  |  |  |  |  |  | - | rho=0.7538  *P*<0.0001 | rho=0.6489  *P*<0.0001 | rho=0.6466  *P*<0.0001 |
| IL-12 |  |  |  |  |  |  |  |  |  |  |  |  | - | rho=0.9085  *P*<0.0001 | rho=0.9083  *P*<0.0001 |
| *Cldn1* |  |  |  |  |  |  |  |  |  |  |  |  |  | - | rho=0.998  *P*<0.0001 |

***NS**: non-significant

**Supplementary Table 4: Correlations between putative HCC predictors and clinicopathological markers in non-cirrhotic patients (*n*=133)**

|  | Age | Child-Pugh | AFP | ALT | AST | BiL | ALB | FIB-4 | PLT | INR | sCD163 | IL-10 | IL-12 | *Cldn1* | *TGF-β* |
| --- | --- | --- | --- | --- | --- | --- | --- | --- | --- | --- | --- | --- | --- | --- | --- |
| Age | - | *NS | rho=-0.1752  *P*=0.0437 | NS | NS | NS | NS | rho=0.5243  *P*<0.0001 | rho=0.2115  *P*=0.0145 | rho=-0.175  *P*=0.0439 | NS | rho=-0.2074  *P*=0.0147 | rho=-0.2096  *P*=0.0155 | NS | NS |
| Child-Pugh |  | - | NS | NS | NS | NS | NS | NS | NS | NS | NS | NS | NS | NS | NS |
| AFP |  |  | - | rho=-0.1841  *P*=0.0339 | rho=-0.2009  *P*=0.0204 | NS | NS | rho=-0.2414  *P*=0.0051 | NS | rho=0.8924  *P*<0.0001 | rho=-0.375  *P*<0.0001 | rho=0.9753  *P*<0.0001 | rho=0.9704  *P*<0.0001 | rho=0.6473  *P*<0.0001 | rho=0.6439  *P*<0.0001 |
| ALT |  |  |  | - | rho=0.8192  *P*<0.0001 | NS | rho=0.5584  *P*<0.0001 | rho=0.4363  *P*<0.0001 | rho=-0.2709  *P*=0.0016 | NS | rho=0.2861  *P*=0.0008 | NS | NS | rho=0.256  *P*=0.0029 | rho=0.2443  *P*=0.0049 |
| AST |  |  |  |  | - | NS | rho=0.7819  *P*<0.0001 | rho=0.6431  *P*<0.0001 | NS | rho=-0.1832  *P*=0.0348 | rho=0.2529  *P*=0.0033 | rho=-0.1922  *P*=0.0278 | NS | rho=0.2375  *P*=0.0059 | rho=0.2241  *P*=0.0101 |
| BiL |  |  |  |  |  | - | NS | NS | NS | NS | NS | NS | NS | NS | NS |
| ALB |  |  |  |  |  |  | - | rho=0.5759  *P*<0.0001 | NS | NS | NS | NS | NS | rho=0.4613  *P*<0.0001 | rho=0.4486  *P*<0.0001 |
| FIB-4 |  |  |  |  |  |  |  | - | rho=-0.3587  *P*<0.0001 | rho=-0.2099  *P*=0.0153 | NS | rho=-0.2633  *P*=0.0024 | rho=-0.2462  *P*=0.0043 | NS | NS |
| PLT |  |  |  |  |  |  |  |  | - | NS | NS | NS | NS | NS | NS |
| INR |  |  |  |  |  |  |  |  |  | - | rho=-0.315  *P*=0.0002 | rho=0.9227  *P*<0.0001 | rho=0.9174  *P*<0.0001 | rho=0.4812  *P*<0.0001 | rho=0.4736  *P*<0.0001 |
| sCD163 |  |  | | | | | | | | | - | rho=-0.337  *P*<0.0001 | rho=-0.312  *P*<0.0001 | rho=-0.4605  *P*<0.0001 | rho=-0.4651  *P*<0.0001 |
| IL-10 |  |  |  |  |  |  |  |  |  |  |  | - | rho=0.9772  *P*<0.0001 | rho=0.598  *P*<0.0001 | rho=0.5932  *P*<0.0001 |
| IL-12 |  |  |  |  |  |  |  |  |  |  |  |  | - | rho=0.6078  *P*<0.0001 | rho=0.6036  *P*<0.0001 |
| *Cldn1* |  |  |  |  |  |  |  |  |  |  |  |  |  | - | rho=1.000  *P*<0.0001 |

***NS**: non-significant
